# Stop Reproducing the Reproducibility Crisis

**DOI:** 10.1523/ENEURO.0032-23.2023

**Published:** 2023-02-03

**Authors:** Christophe Bernard

Crisis? What crisis? Is the lack of reproducibility a supertramp that disperses widely across science fields? Is it carelessness, or is it also intrinsic to life sciences as I shall argue? Lack of reproducibility is a big flag brandished by many, but what do they mean by it? Where does it originate from?

These were the first questions that crossed my mind as, while in Liège, Belgium for a PhD defense, I was asked to give a talk on the reproducibility crisis. Rather than a formal presentation, we had an open discussion. This editorial is its outcome.

According to Wikipedia’s definition of reproducibility, “results obtained by an experiment or an observational study or in a statistical analysis of a data set should be achieved again with a high degree of reliability when the study is replicated” (https://en.wikipedia.org/wiki/Reproducibility). The definition is thus very strict: the results must be replicable, which means that the same experimental conditions or data analysis method must be used. Finding similar results with different approaches is not replicability *stricto sensu*; it just gives more weight to a study and equates to generalizability. Most often there is a confusion between reproducibility of the conclusions and reproducibility of results.

## Reproducibility of Conclusions Is Not Reproducibility of Results

Modern neuroscience sometimes requires the development of homemade tools or the use of very expensive equipment. Consequently, some results cannot be replicated using the same experimental conditions. If we cannot perform the original experiments, we design different ones with the aim of obtaining the same conclusions. If we cannot obtain the same conclusions, it is tempting to conclude that it is a failure to reproduce. But failing to reproduce the conclusions does not mean that the original study was flawed.

Failing to reproduce the conclusions is expected in science. It is even necessary to spawn discussions and for a field to move forward. This is what the philosophy of science has taught us for centuries. Otherwise, we would still adhere to Aristotle’s conclusion that the brain is a cooling system for the blood. Reaching different conclusions does not contribute to the reproducibility crisis.

In modern science, the confusion between results and conclusions is constant. Why? There are so many papers to read that it has become impossible to remember all the details of the experimental procedures. Indeed, we tend to only remember one thing: the main conclusion of the paper, usually its title, and rarely the observations themselves. While the observations are factual and quantifiable, the conclusions cannot be quantified. Therefore, it is important for young scientists to realize that they need to have a very thorough knowledge of a restricted number of papers with all their experimental details, to build a rationale for their study. Starting from the conclusions would be a mistake.

## Conclusions Are Questionable Because There Is No Generic Theory of the Brain

An experiment produces results, which we quantify—these are facts. Then, the results are interpreted, usually in the context of an underlying hypothesis. Hypotheses are themselves embedded in a conceptual framework underlying the system we are studying. In neuroscience, we do not have a general theory of the brain. While physics is bounded by the relativity theories (special and general relativity theories led to predictions, which were verified experimentally), neuroscience is an unbound field, and consequently, we are building our ideas of brain function as knowledge accumulates. Our conceptual framework is constantly changing over time. This is why some results obtained decades ago are reinterpreted repeatedly as our understanding progresses. Since conclusions derive from the interpretation of the observations, hence the conceptual framework that is used, the validity of conclusions is (and must be) questionable. By the very construction of the scientific method, failure to reproduce conclusions is expected; it should not be vilified. In a way, it is necessary.

The solution is straightforward: we scientists should be more careful when drawing strong conclusions because, once they reach the public, e.g., “targeting Aβ plaques will cure Alzheimer’s disease,” they produce strong expectations. Failure to deliver is often wrongly associated with reproducibility crisis, because what is retained is the (sometimes overstated) conclusion of the study, not what the actual results are. In addition, conclusions, but not results, belong to rhetoric, with its three main aspects: logos (logic and reason), ethos (reputation of the lab), and pathos (emotions, particularly for diseases). A nicely crafted conclusion, or one that appeals to scientists, will convince them even if there are weak supporting facts. A very important part of the education of young researchers must therefore be to disentangle actual results from interpretations and to evaluate the distance between the two. In other words, how close are the conclusions from the data?

Since reproducibility is not a concept that applies to conclusions, what can we say about the alleged crisis regarding the reproducibility of results?

## The Main Lesson in Biology: Results Are Inherently Difficult to Reproduce

A gold standard of reproducibility may help us defining expectations and goals to achieve in neuroscience. I am aware of only one field where reproducibility is the norm: mathematics. Mathematics (including algebra, geometry, calculus, and analysis) is based on fundamental truths and axioms. When someone correctly demonstrates a theorem, the demonstration and its results can be repeated by other mathematicians.

We do not yet have fundamental truths in biology. However, there is a central axiom: degeneracy. Degeneracy is the multiplicity of solutions to converge to a given output or function (Rathour and Narayanan, 2019). Let us take the example of the construction of a bursting neuron with 10 possible ion channel species. One may end up with multiple ways (or solutions) to combine these 10 channels while varying their properties to generate the same bursting behavior. If we consider all solutions, we obtain for each channel a more or less wide distribution of possible values. When we perform actual measurements of a given channel in real neurons of a given type, we observe different values, not only across animals but also within the same animal. This variability does not result from the imprecision of the measure (although the latter contributes to it) but rather reflects degeneracy. Considering degeneracy as a core principle in biology provides a rational explanation to the failure to replicate. If the parameter you are measuring has a wide distribution in the set of solutions, two samples obtained from this distribution may provide very different values (see the “dance of the *p* values”; https://www.youtube.com/watch?v=3FjoUOolOYQ). Although different, both represent accurate descriptions of biological reality.

Let us now consider a preclinical study testing a drug for a given disease. This drug targets the ion channel previously mentioned as having a wide distribution. If the test is performed on a set of animals in which the channel is highly expressed, there will be a strong effect. Another lab now makes the same test but uses animals from the same strain with a low level of expression (which is statistically possible): there will be no effect of the drug.

Although, *stricto sensu*, it is a failure to replicate, obtaining different results may just reveal the intrinsic variability that characterizes (all?) living organisms. I argue that the concept of replicability poorly applies to life sciences because it negates the core property of life: diversity. This is what pharmacotherapy teaches us every day. Some patients respond positively to a given treatment, some see no change, some get worse. Universality poorly applies to living organisms.

Of course, the extent of degeneracy is variable across biology. Isoprenaline increases heart frequency and inotropism. Caffeine increases vigilance and diuresis. Opiates induce constipation. Why some functions are so determined by few biological actors (and thus have little inbuilt variability) and others lend themselves to degeneracy is unknown.

Life sciences have been influenced too strongly by exact sciences, such as astronomy, theoretical physics, etc. In the latter, universal rules are assumed to exist, and they can be verified experimentally. Carbon ^12^C is made of six protons, six neutrons, and six electrons. The 15 other known carbon isotopes, like ^14^C, contain more or fewer neutrons, but they have different properties. There is only one way to make a carbon atom with the properties of ^12^C. You cannot change the number of protons, electrons, and neutrons without changing the properties of the atom. In neurons, you can change the properties of its building blocks and still obtain the same behavior.

Over the years, we have tried to standardize research. We use the same inbred species and strains. Animals are raised in controlled pathogen-free conditions, etc. This gives a false impression that results should be replicable, but unfortunately this is not the case. Animals are not genetic clones, and epigenetic mechanisms and life experiences make them diverge. Behavioral studies always require many animals because animals are biologically different, even if they come from the same litter (Manouze et al., 2019).

Caution should be exerted when claiming failure to replicate. Degeneracy should always be considered as a possible explanation. Yet, failures to replicate have more prosaic and well-known causes.

## Ego and Carelessness Are Intrinsically Embedded in the Replicability Crisis

The way the system dysfunctions is summarized as: publish in “high” impact journals or perish. In most cases, where and how often you publish will be used as criteria to decide whether you will be accepted in a lab to do a postdoc, whether you will be recruited or promoted, and whether you will get a grant. This pressure, consciously or nonconsciously, may drive some scientists to obtain the results that make a great story, support a theory, etc. During private discussions, several students and postdocs confided in me: “I cannot publish my results as long as they do not fit with what the PI wants,” or “I had to select 25% of the results that fit with the theory.” These are infrequent cases, fortunately, but they do occur. Knowing what the results *should* be may also drive some to unconsciously tune the experimental conditions with that result in view.

The only solution is to be trained in scientific rigor and flee any lab where such practices take place.

The way statistics is performed is also a major contributor to the replicability crisis. Many papers have been written on the misuse of statistics (such as *p* hacking). As mentioned above, our measurements consist in sampling a distribution of possible values that can be very wide. A *p* value is informative, but it must not be considered as an absolute truth, it is merely a probability. Since we have no idea about the size of the real distribution we are sampling from, calculating the confidence interval provides important information (see Alger, 2022; Calin-Jageman, 2022). Whether you are a frequentist or a Bayesian, a true understanding of what statistical values mean should make us more careful regarding the conclusions we reach. Also being able to calculate the effect size of a manipulation and discuss it adequately should be an important part of a paper. There is a growing number of very interesting efforts in this area, e.g., Figure 6 in Ganguly et al. (2021). Training students as early as possible in all these aspects is clearly the solution.

The hypothetico-deductive model is at the core of the scientific method that we are using. It starts with formulating a hypothesis that is falsifiable, and then test it. However, many studies fall in the trap of fallacies (Bernard, 2020), especially the most common: affirming the consequent. A classical one is: if I am experiencing fear during a functional MRI session, then area X will “light up.” This is the observation. Affirming the consequent is: if area X “lights up” in MRI, therefore I am experiencing fear. Although this relates more to the failure to replicate the conclusions because of a lack in scientific logic; the way the experimental procedure is built can be based on fallacies (mostly ignoring alternate explanations), which could lead to results that cannot be reproduced. For example, we have the hypothesis that area X is coding for fear. We design an experiment during which we show fearful faces to test subjects while they are in the MRI machine. We observe that X “lights up,” and we conclude that X codes for fear. Although this is possible (provided that we test all other possibilities that could lead to an activation of X), the experiment only shows that if I experience fear, I see an activation of X. Many experiments are designed similarly. I hypothesize that protein Y is necessary for synaptic plasticity. If I knock out gene X, I observe that synaptic plasticity cannot be triggered anymore. Despite the latter observation, it is not possible to conclude that Y is necessary for synaptic plasticity. An alternate explanation can be that removing Y alters cell metabolism, and that there is not enough ATP for synaptic plasticity-dependent mechanisms. The same line of reasoning is used in numerous experiments using cell/network manipulation to claim causality regarding function, including optogenetics and chemogenetics. These examples show that it is very easy to fall into the trap of generalization and fallacies. To quote Claude Bernard, we should remain the slave of the observation. Here again, education in scientific logic is key.

## Stop Reproducing the Reproducibility Crisis

In conclusion, if misdeeds have always existed in science, failure to reproduce mostly stems from confusing replicating conclusions and replicating results, not accounting for degeneracy, and lack of education in statistics and scientific logic. How can we stop it? By being more careful when interpreting results and drawing conclusions, and by educating. In this assay, I have quickly brushed a vast topic. Anyone interested in contributing to our collective thinking on reproducibility is welcome to contact me.

I wish to express my thanks to members of the scientific community of Liège University for the lively discussion we had on the topic.
